# The X-ray Pump–Probe instrument at the Linac Coherent Light Source

**DOI:** 10.1107/S1600577515005135

**Published:** 2015-04-21

**Authors:** Matthieu Chollet, Roberto Alonso-Mori, Marco Cammarata, Daniel Damiani, Jim Defever, James T. Delor, Yiping Feng, James M. Glownia, J. Brian Langton, Silke Nelson, Kelley Ramsey, Aymeric Robert, Marcin Sikorski, Sanghoon Song, Daniel Stefanescu, Venkat Srinivasan, Diling Zhu, Henrik T. Lemke, David M. Fritz

**Affiliations:** a Linac Coherent Light Source, SLAC National Accelerator Laboratory, 2575 Sand Hill Road, Menlo Park, CA 94025, USA; b Paul Scherrer Institut, CH-5232 Villigen, Switzerland

**Keywords:** FEL, X-ray, pump–probe, time-resolved

## Abstract

The X-ray Pump–Probe instrument achieves femtosecond time-resolution with hard X-ray methods using a free-electron laser source. It covers a photon energy range of 4–24 keV. A femtosecond optical laser system is available across a broad spectrum of wavelengths for generating transient states of matter. The instrument is designed to emphasize versatility and the scientific goals encompass ultrafast physical, chemical and biological processes involved in the transformation of matter and transfer of energy at the atomic scale.

## Abstract 2

A description of the X-ray Pump–Probe (XPP) instrument at the Linac Coherent Light Source. is presented. Recent scientific highlights illustrate the versatility and the time-resolved X-ray diffraction and spectroscopy capabilities of the XPP instrument.

## 1. Introduction   

The Linac Coherent Light Source (LCLS), a US Department of Energy Office of Science user facility operated by Stanford University, achieved first light in 2009 (Emma *et al.*, 2010 ▶). The X-ray Pump–Probe (XPP) instrument, located in the third hutch of the Near Experimental Hall (NEH), was the first hard X-ray instrument to receive LCLS hard X-rays and started user operation in 2010. LCLS provides transversely coherent hard X-rays with unprecedented flux and short pulse duration. These characteristics enable the experimental investigation of structural dynamics with X-ray techniques by directly following the time evolution of the electron density during the course of a photo-induced biological, chemical or physical transformation. The XPP instrument predominantly uses ultrafast optical laser pulses to generate transient states of matter. Hard X-ray pulses from the LCLS probe the structural dynamics initiated by the optical excitation. The optical laser has the ability to create or access a wide variety of excited states, and these can be manipulated by changing the laser pulse energy, wavelength, temporal profile and even using various nonlinear excitation mechanisms. X-ray absorption and emission spectroscopy are used to track changes in the electronic and site-specific nuclear structure, and X-ray scattering is the dominant tool for probing concomitant structural changes.

## 2. Instrument overview   

The XPP instrument operates in the hard X-ray regime (*i.e.* above 4 keV). A summary of the XPP instrument parameters is listed in Table 1 ▶. A set of mirrors, consisting of silicon-carbide coated silicon substrates with an incidence angle of 1.32 mrad, is located in the front-end enclosure of the NEH which feeds the hard X-ray beam to all LCLS hard X-ray hutches. These mirrors also serve to define the maximum photon energy that can be delivered to the LCLS hard X-ray hutches to 25 keV. The fundamental photon energy covers the 4–11 keV range; higher photon energies can be reached by using the third harmonic.

**Figure 1:**
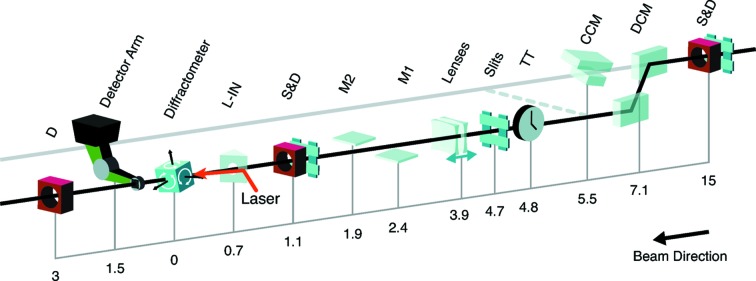
Overview of the XPP instrument layout. Distances are indicated in meters from the center of the diffractometer. S&D are slits and non-destructive intensity diagnostics, DCM is a large-offset double-crystal monochromator, CCM is a channel-cut monochromator, TT is a timetool measuring the arrival time of the optical laser in reference to the X-rays, M1 and M2 are silicon mirrors that can be used to deflect the beam in the vertical direction and can also provide harmonic rejection, L-IN is the laser in-coupling for the optical laser, D is a diagnostic. Components located downstream of the dashed line can be translated into the main LCLS line and allow the XPP instrument to take advantage of the full power and properties of the fundamental. The gray line is the LCLS main line, whereas the black one is offset 600 mm horizontally by the DCM. The sample at the XPP instrument is located approximately 200 m downstream of the undulators.

The most upstream components of the XPP instrument are located in hutch 2 and consist of slits and diagnostics located 15 m upstream of the sample as indicated in Fig. 1 ▶. The X-ray beam enters hutch 3, the XPP hutch, just before the double-crystal monochromator, indicated in Fig. 1 ▶ by DCM. We now further describe the specifications of the key components of the XPP instrument.

*Monochromators.* The XPP instrument has two distinct configurations. It can operate in the LCLS main line and can therefore take full advantage of the first-harmonic capabilities of the LCLS (SASE, seeding, two-color, *etc*.) (Amann *et al.*, 2012 ▶; Lutman *et al.*, 2014 ▶). To operate in this configuration, all components located downstream of the dashed line in Fig. 1 ▶ are translated into the LCLS main line (indicated by the gray line in Fig. 1 ▶). In that configuration a Si(111) channel-cut monochromator (CCM), operating in vertical scattering geometry (Narayanan *et al.*, 2008 ▶), allows a narrower bandwidth (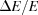
= 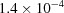) to be delivered to experiments that require broad and intensive scans of the incident photon energy.

For experiments that require a monochromatic beam, but do not intend to scan the incident energy significantly, a custom-built (JJ X-ray, Denmark) large-offset DCM (Zhu *et al.*, 2014 ▶) is used. In that configuration, all components located downstream of the dashed line are translated 600 mm horizontally as displayed in Fig. 1 ▶. The DCM can either operate with Si(111) or C*(111) crystals. When operating with the latter, it not only provides a better energy resolution (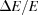 = 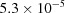) but most importantly allows for multiplexing with another instrument located downstream in the Far Experimental Hall (Feng *et al.*, 2013 ▶; Zhu *et al.*, 2014 ▶, 2015 ▶). When operating with the Si(111), only XPP can use the LCLS beam when the crystal is in place. Fortunately, the beam can be rapidly provided to any of the FEH instruments by translating the first silicon DCM crystal from the beam path.

*X-ray focusing lenses.* The unfocused beam at XPP is typically 0.5 mm × 0.5 mm in size. Beryllium compound refractive lenses can focus the beam (Snigirev *et al.*, 1996 ▶) and thereby provide beam size control in one or two dimensions. The lenses are located 3.85 m upstream of the sample and can be adjusted ±150 mm longitudinally. The minimum spot size was measured to be slightly below 2 µm (as a result of the finite SASE bandwidth and optics imperfections). An asymmetric focusing option allows a line focus to be produced in the sample plane for applications such as grazing-incidence experiments or elongated samples.

*Pulse picker.* A fast shutter is available for selecting single X-ray pulses from the LCLS pulse train on demand, as well as for reducing the repetition rate. It consists of a rotating channel whose angle is swung back and forth by a stepper motor to create a brief opening time. It can be used to create arbitrary pulse train time patterns provided that the pulse train structure has an average rate of equal to or less than 10 Hz.

*Mirrors.* Two silicon mirrors located 1.9 and 2.4 m upstream of the sample allow the beam to be delivered with a vertical grazing angle (as, for example, required for liquid surfaces). They can also offer the interesting option to reduce the third-harmonic content of the LCLS beam by operating them above the critical angle for these energies.

*Diffractometer.* A custom-built (Huber, Germany) diffractometer provides various degrees of freedom together with high load capacity, thus allowing the precise orientation of samples or sample environments such as vacuum chambers, gas/liquid injectors, *etc*. A kappa diffractometer module is also available. The detector positioning is provided by a robotic arm (Stäubli, Switzerland). It provides a sample–detector distance of up to 1.5 m and can either operate in cartesian or spherical coordinates once the origin is defined (*i.e.* sample).

*Optical laser system.* Core laser systems at the LCLS consist of an ultrashort-pulse Ti:sapphire oscillator synchronized to the FEL seeding a commercially available chirped pulse amplifier producing 4 mJ at 40 fs. An additional home-built four-pass amplifier can boost the pulse energy to over 30 mJ to most of the experimental hutches. Wavelength conversion inside the experimental hutches can cover a broad spectral range from 200 nm to 150 µm. A more in-depth description of the optical laser capabilities at LCLS is given by Minitti *et al.* (2015 ▶).

*Timing diagnostics.* Typical phase locking between the accelerator and the laser system only hold the temporal jitter between the two sources to about 200 fs full width at half-maximum (FWHM). In order to take full advantage of the short pulses and reach pulse-length-limited time resolution, diagnostics that measure the relative arrival time between laser and X-ray pulses have been developed. After demonstrating both a spatial and spectral approach for such a diagnostics for hard X-rays at XPP (Harmand *et al.*, 2013 ▶), the spectral method was implemented into a standard beamline diagnostics (Bionta *et al.*, 2011 ▶; Lemke *et al.*, 2013 ▶). It is based on the X-ray induced change in refractive index of a thin target probed by a chirped broadband white-light continuum pulse derived from the optical laser. The X-ray induced change of optical light transmission can be resolved in an optical spectrometer for each pulse. Target materials used are typically either silicon nitride (Si_3_N_4_) or Ce:YAG crystals of different thicknesses, to accommodate for different beam conditions.

*Additional diagnostics.* The self-amplified spontaneous emission (SASE) process induces pulse-to-pulse fluctuations of the beam properties, such as pulse energy, duration, spatial profile, wavefront, temporal profile and spectral content. *In situ* pulse property monitoring is thus crucial for data interpretation. Multiple intensity–position monitors (Feng *et al.*, 2011 ▶) are installed at various locations along the instrument for pulse-to-pulse intensity normalization. The spatial profile of the LCLS beam can be also measured at various locations along the XPP instrument using scintillating screens with high-resolution camera-lens combinations.

*Spectrometers.* The spectral intensity profile can be measured on every shot by means of a thin (bent) crystal transmissive spectrometer (Zhu *et al.*, 2013 ▶). This device has an energy range that covers the full X-ray FEL spectrum and sufficient resolution to resolve individual SASE spikes. A multi-crystal wavelength-dispersive X-ray emission spectrometer based on the von Hamos geometry and a monochromatic analyser crystal based on the Rowland geometry are also available for photon-in photon-out spectroscopic measurements (Alonso-Mori *et al.*, 2012 ▶).

*Detectors.* Several X-ray detectors are available and integrated with the XPP data acquisition system: the 1 Mpixel CSPAD, the 140 kpixel CSPAD-140k (Herrmann *et al.*, 2013 ▶), a Rayonix MX170-HS detector, and the ePix series detector under development (Dragone *et al.*, 2014 ▶). For more information about the LCLS detectors see Blaj *et al.* (2015 ▶) ▶.

## 3. Highlights   

The XPP instrument has been used in a wide range of scientific investigations ranging from materials science, chemistry, nonlinear optics and protein crystallography. The following examples illustrate some capabilities of the instrument.

### 3.1. Femtosecond diffraction   

Femtosecond diffraction has been envisioned as one of the primary measurement techniques at the XPP instrument to elucidate complex interactions and correlations in materials *via* the study of transient atomic scale structural dynamics generated by ultrafast excitations. For example, Beaud *et al.* (2014) ▶ observed the ultrafast melting of the charge and orbitally ordered phase in an epitaxically grown perovskite manganite film [Pr_0.5_Ca_0.5_MnO_3_ (PCMO)]. The film was pumped with a 55 fs, 800 nm optical pulse focused down to 230 µm × 230 µm. By taking advantage of the intense and short X-ray pulses, different superlattice reflections, each sensitive to different components of the phase transition, were measured.

**Figure 2:**
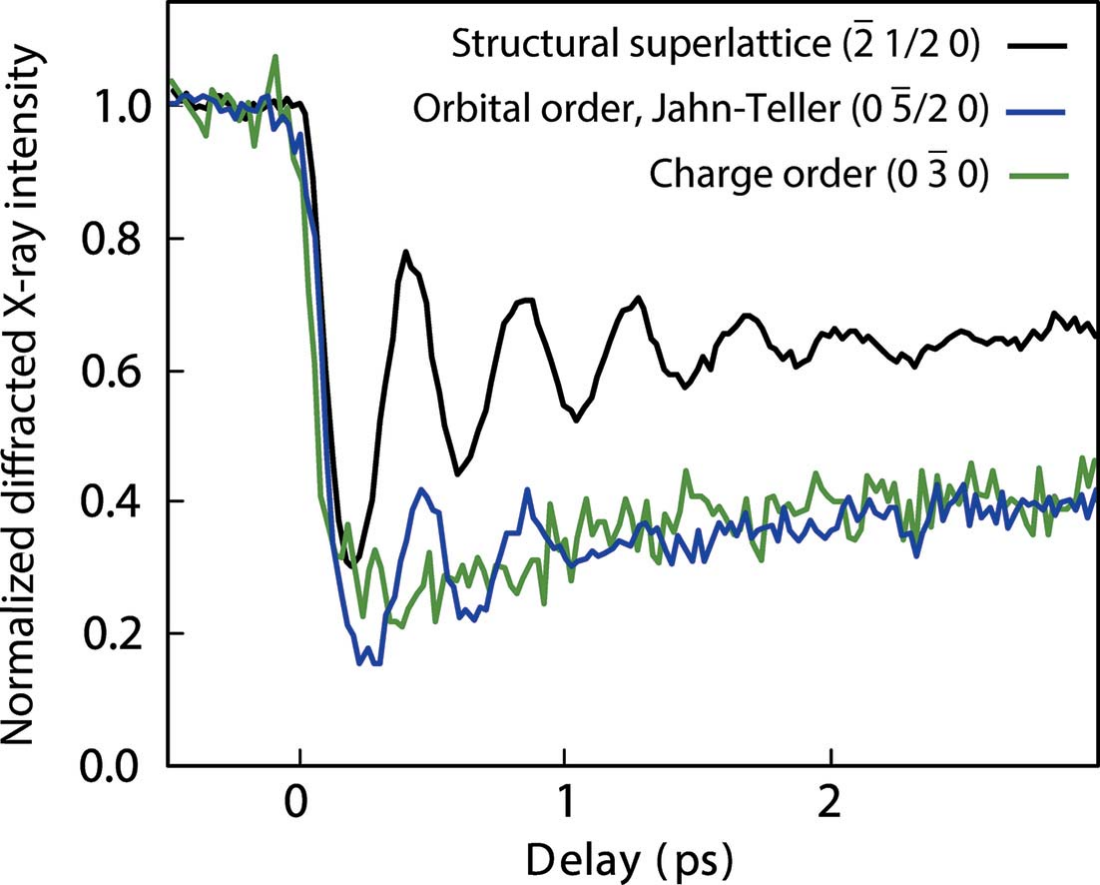
Time-resolved normalized diffracted X-ray intensity for three superlattice reflections. The (−2 1/2 0) reflection is measured off resonance at 6.53 keV. The (0 −5/2 0) reflection is measured near resonance at 6.553 keV, and the (0 −3 0) reflection is measured on resonance at 6.555 keV. [Reprinted by permission from Macmillan Publishers Ltd: Beaud*et al.* (2014 ▶). *Nat. Mater.* **13**, 923–927, copyright (2014).]

Reflections (0 *k*/2 0) are sensitive to the orbital order and Jahn–Teller distortion, while (0 *k* 0) reflections are more sensitive to the charge order. Reflections (*h* *k*/2 *l*) give information about the overall structural distortion. Fig. 2 ▶ shows the observed time-resolved signal for different superlattice reflections at a pump fluence of 2.7 mJ cm^−2^. The observed dynamics are dominated by a 2.45 THz oscillation which is characteristic of a coherent optical phonon mode in those type of materials when excited with short optical pulses (Matsuzaki *et al.*, 2009 ▶).

In another experiment using femtosecond diffraction, Först *et al.* (2013) ▶ and Mankowsky *et al.* (2014) ▶ demonstrated the potential of using photoexcitation to drive phase transitions in strongly correlated material systems. More generally, the ability to measure lattice dynamics with femtosecond X-rays opens the way towards better understanding and control of ultrafast phase transitions.

### 3.2. Femtochemistry   

Femtochemistry of molecular sample systems in solution is another field of research present at the XPP instrument. The ultrafast timescales accessible at LCLS allow for the first time to investigate chemical processes like charge transfer, bond cleavage and molecular vibrations in a solvated environment. These processes are highly relevant for applied chemical or biological processes. A large interest is devoted to molecular photocatalysts due to their potential for charge separation, which can technically be exploited for energy conversion from optical light. In that context, dissolved iron(II) tris(2,2′-bipyridine) ([Fe^II^(bpy)_3_]^2+^) has been studied intensively at XPP, as a model system for abundant transition metal complexes. Here, an optically excited metal-to-ligand charge transfer (MLCT) state is depleted within less than 200 fs to a high-spin (HS) state, which has been previously extensively studied by optical and X-ray methods (Chergui, 2013 ▶; Canton *et al.*, 2014 ▶; Gawelda *et al.*, 2007 ▶; Bressler *et al.*, 2009 ▶).

**Figure 3:**
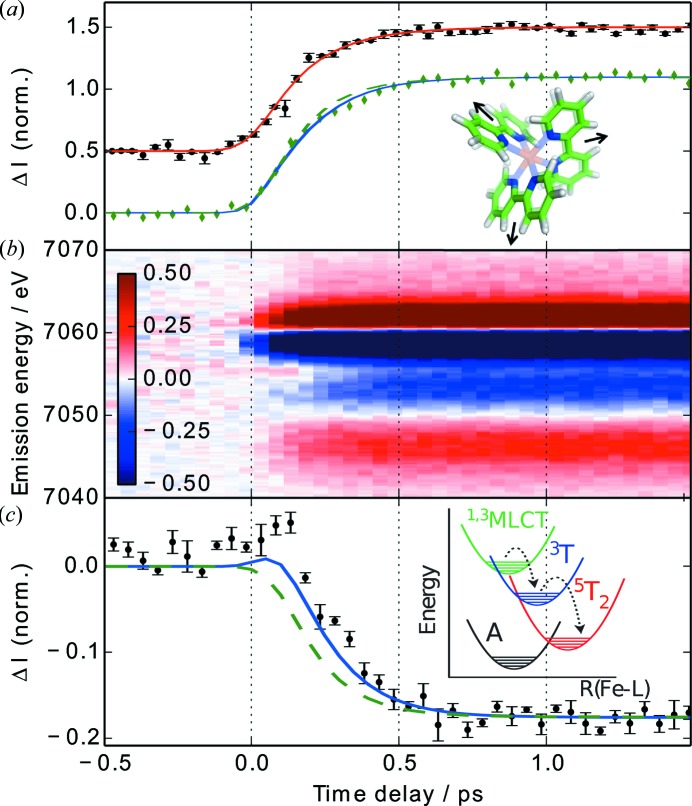
Spectroscopic pump–probe results from the photoexcited spin transition of [Fe^II^(bpy)_3_]^2+^ in solution. After excitation into the MLCT state, transition into the HS ^5^T_2_ state [inset (*c*)] causes an increase of the Fe-to-ligand distance [inset (*a*)]. This structural change is tracked by the XANES trace at 7125 eV [(*a*), black symbols, vertically offset by 0.5]. The structural HS rise has been fitted by an exponential rise of 162 ± 6 fs (Lemke *et al.*, 2013 ▶). (*b*) Time-dependent *K*β emission difference spectra. The transient traces at 7061 eV and 7054 eV have been extracted [(*a*) green diamond symbols and (*c*) black symbols, respectively]. The data have been overlaid with results from global fits including (blue lines) and excluding (green dashed lines) population of and intermediate ^3^T triplet state (Zhang *et al.*, 2014 ▶). [Reprinted by permission from Macmillan Publishers Ltd: Zhang *et al.* (2014) ▶. *Nature (London)*, **509**, 345–348, copyright (2014).]

Optically induced local structural changes around the Fe center of the molecule were studied through changes in the Fe *K*-edge X-ray absorption near-edge structure (XANES) (Lemke *et al.*, 2013 ▶). An aqueous [Fe^II^(bpy)_3_]^2+^ solution in a liquid sheet jet was excited to the MLCT state and the transition cascade into the HS state consequently probed by changes to the absorption cross section of monochromatic X-rays. The intense FEL pulses allowed the entire transient XANES spectra to be scanned in minutes with sufficient statistics to resolve the transient changes. The observed pump–probe difference spectra confirmed picosecond results from the HS state recorded at a synchrotron source (Gawelda *et al.*, 2007 ▶), while at short time delays a hint of a different spectral component, potentially the MLCT state, was observed. The ultrafast rise of the HS state XANES signals could for the first time be characterized (Fig. 3*a* ▶). More detailed information about the transient electronic transitions in [Fe^II^(bpy)_3_]^2+^ were obtained by X-ray emission spectroscopy (Zhang *et al.*, 2014 ▶).

Fluorescence lines from occupied electron levels to core holes created by the X-rays contain information about the chemically active electron configurations. In iron, the *K*β_1,3_ emission line reflects changes of the Fe 3*d* electron spin state through interaction of 3*d* and 3*p* electrons (Glatzel & Bergmann, 2005 ▶) while it remains less sensitive to changes of the chemical configuration (Lee *et al.*, 2010 ▶). Transient combinations of spin state can therefore be interpreted through ground-state reference spectra. Reference samples with similar coordination bonding were chosen in order to account for the remaining covalent content in the *K*β_1,3_ spectra. An energy-dispersive von Hamos spectrometer populated with four cylindrically bent Ge(620) crystal analyzers (Alonso-Mori *et al.*, 2012 ▶) was used to measure the entire *K*β_1,3_ line with a CSPAD-140K area detector. The changes in the emission lines were recorded and sorted as a function of pump–probe time delay (Fig. 3*b* ▶). Analysis using reference spectra revealed experimental proof for population of a ^3^T triplet state as intermediate state between MLCT and HS state (Fig. 3*c* ▶), which had previously been debated on the basis of ultrafast XANES measurements (Bressler *et al.*, 2009 ▶).

## 4. Conclusion   

The XPP instrument is a versatile tool for performing pump–probe experiments in the hard X-ray regime. The combination of the large number of photons per pulse and the temporal resolution characteristic of LCLS allows for investigation of ultrafast physical, chemical and biological processes. X-ray scattering, absorption and emission spectroscopy are some of the dominant techniques used at the XPP instrument for probing structural and electronic changes. More details about the XPP instrument can be found on the following website: http://lcls.slac.stanford.edu/xpp. 

## 5. Facility access   

LCLS instruments are open to academia, industry, government agencies and research institutes worldwide for scientific investigations. There are two calls for proposals per year and an external peer-review committee evaluates proposals based on scientific merit and instrument suitability. Access is without charge for users who intend to publish their results. Prospective users are encouraged to contact instrument staff members to learn more about the science and capabilities of the facility, and opportunities for collaboration.

## Figures and Tables

**Table 1:** X-ray parameters and capabilities of the XPP instrument

Instrument name	XPP
Mirrors, incidence angle	2 SiC on Si, 1.32mrad
Monochromaticity ( 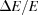 )†	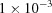 (SASE), 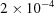 (seeding)
Energy range (keV)	4 to 11 (fundamental)
Unfocused beam size (m)	500 at 8.3keV
Focused beam size (m)	2500
Focusing capability	Be lenses, 1D and 2D focusing
Flux (photons pulse^1^)	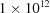 (fundamental‡)
Pulse length (fs)	5200
Repetition rate	120, 60, 30, 10, 5, 1, on demand
Optical laser pulse energy (mJ)	20 (800nm), 45 (400nm), 1 (266nm)
Optical laser pulse width (fs)	10150
Standard detectors	CSPAD, CSPAD-140K, ePix, Rayonix, ORCA, PIPS diodes
Sample environment	Huber three-circle goniometer, kappa diffractometer, general purpose vacuum chamber, liquid jet, He enclosure, Oxford LN_2_ cryojet down to 100K

† Typical single-shot value.

‡ Excluding beamline and instrument transmission.
